# Molecular bases of neurophysiologic dysfunctions in migraine

**DOI:** 10.1186/1129-2377-16-S1-A15

**Published:** 2015-09-28

**Authors:** Cherubino Di Lorenzo

**Affiliations:** Don Carlo Gnocchi Onlus Foundation, Rome, Italy

In past years, several studies evidenced neurophysiologic dysfunctions in sporadic episodic (during both the ictal and the interictal period), and chronic migraine patients. These dysfunctions could reflect a genetic susceptibility that exposes patients to recurrence of migraine attacks.

Recently, several studies were performed in order to explore the role of genetic polymorphisms on neurophysiologic dysfunctions that were already known to be related to migraine. These studies evidenced that neurophysiologic features, such as interictal habituation deficit and ictal response sensitization, are influenced by genetic polymorphisms involved in neural plasticity (brain derived neurotrophic factor [BDNF]), in vascular homeostasis (angiotensin converting enzyme [ACE] and methylenetetrahydrofolate reductase [MTHFR]), and in monoaminergic modulation (monoamine oxidase type A [MAO-A]). Moreover, we have recently found that a glutamatergic polymorphism, Glutamate receptor ionotropic AMPA 3 (GRIA3), already known to be related to central sensitization mechanism and then to migraine pathophysiology, can also influence neurophysiologic features of chronic migraine due to medication overuse.

The study of the influence of genetic polymorphisms on migraine-related neurophysiological dysfunctions could be regarded as an “*in vivo*” human experimental model in which the different genetic background could predict different neurophysiologic “endophenotypes”. This experimental model could lead to understand, for instance, the way different drugs act on migraine, or to unveil in which way some risk factors, such as for instance drug overuse, induce transformation from episodic to chronic migraine.

